# Elimination of mutant SWI/SNF complexes by protein quality control: new opportunities targeting aggressive rhabdoid tumours

**DOI:** 10.1038/s41392-024-01935-9

**Published:** 2024-09-06

**Authors:** Andreas Krämer, Stefan Knapp

**Affiliations:** 1 Institute of Pharmaceutical Chemistry, Goethe University Frankfurt, Max-von-Laue-Str. 9, 60438 Frankfurt am Main, Germany; 2 Structural Genomics Consortium, Buchmann Institute for Molecular Life Sciences, Goethe-University Frankfurt, Max-von-Laue-Str. 15, 60438 Frankfurt am Main, Germany; 3 German Cancer Consortium (DKTK), German Cancer Research Center (DKFZ), DTKT Site Frankfurt-Mainz, 69120 Heidelberg, Germany

**Keywords:** Paediatric cancer, Paediatric cancer

A recent study published in *Nature* by Radko-Juettner and colleagues reports an unexpected mutant-specific synthetic lethality in which the E3 protein ubiquitin ligase DCAF5 specifically degrades mutant but not wild-type SWI/SNF chromatin remodeling complexes.^1^ DCAF5 contains a likely druggable WDR domain, providing a new avenue for the development of novel therapeutics for aggressive cancers with SMARCB1 loss of function mutations.

Loss of tumour suppressor function is a common mechanism leading to the development of cancer. Direct targeting of these mechanisms presents a dilemma for drug discovery because the target is lost by a genetic lesion and its function can no longer be restored by a small molecule. The discovery of synthetic lethal relationships that sensitize cancers harbouring loss-of-function mutations has emerged as an attractive strategy for developing targeting strategies but the mechanisms leading to synthetic lethality are often not well understood.^2^ Chromatin remodelling is a highly regulated cellular function that regulates gene expression and the “switching” of genetic programs during cell differentiation and cell state transitions. Remodelling complexes of the SWI/SNF (switch/sucrose non-fermentable) family are multi-subunit proteins containing diverse scaffolding proteins. Three types of SWI/SNF complexes have been identified in humans: BAF (BRG1/BRM-associated factor), PBAF (polybromo-associated BAF) and non-canonical BAF (ncBAF). SWI/SNF complexes contain a catalytic ATPase subunit named SMARCA2 or SMARCA4 that are the ATP driven motor of this remodelling machinery. Recent structural insights provided by cryo-electron microscopy (EM) have revealed the structure of the overall SWI/SNF assembly, the structural basis of nucleosome interaction and the mechanism of histone eviction during the chromatin remodelling process.^3^

The subunit composition of SWI/SNF is a key regulatory mechanism during development and cell state transitions. By exchanging subunits, SWI/SNF complexes control interaction with gene enhancers and promoters, resulting in cell type-specific transcriptional signatures. The subunit encoded by SMARCB1 (SWI/SNF Related, Matrix Associated, actin dependent Regulator of Chromatin, Subfamily B) also called BAF47, is only present in BAF and PBAF complexes but not in ncBAF. Alterations in SMARCB1 expression modulates the balance of between canonical and non-canonical SWI/SNF complexes altering transcriptional signatures by regulating enhancer activity but not super-enhancer activity. Loss of SMARCB1 is a major driver in aggressive synovial sarcoma (SS) based on the presence of a SS18-SSX fusion protein that prevents SMARCB1 interaction with BAF and PBAF. Loss of SMARCB1 is also a genetic hallmark in rhabdoid tumours (RTs) where biallelic inactivation of SMARCB1 is often the sole driver of this aggressive paediatric cancer.^4,5^

**Fig. 1:**
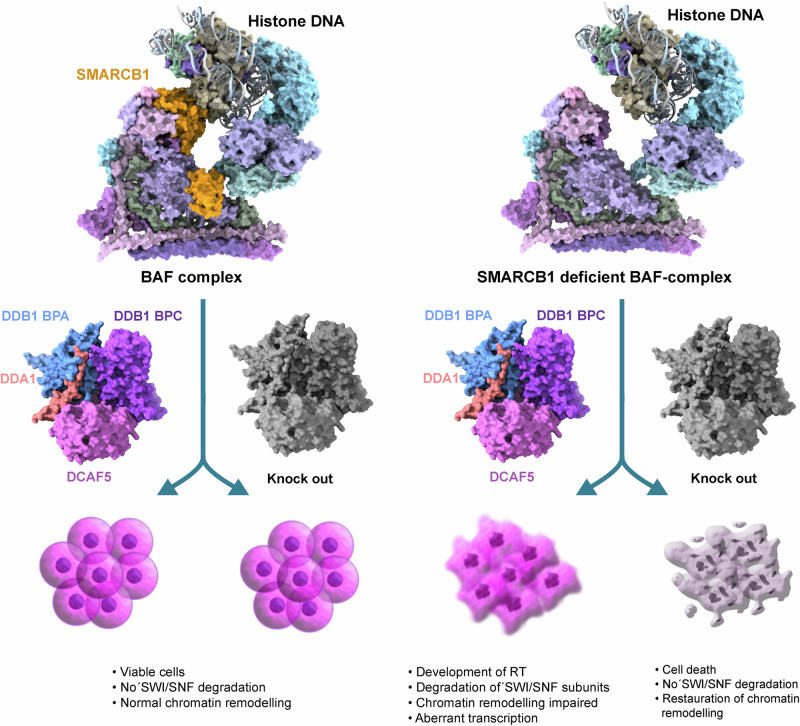
Consequences of DCAF5 depletion in wild type cells (left panel) and in SMARCB1 deficient RT cells (right panel). In RT cells, but not in wild type cells containing functional SWI/NSF remodelling complexes, depletion of DCAF5 leads to cell death and restoration of SWI/SNF remodelling activity (See text for details). The figure has been generated using ChimeraX and Adobe Illustrator

Radko-Juettner et al. used data from 14 SMARCB1-mutant RT cell lines generated from a near-genome-wide CRISPR-Cas9 loss-of-function screen conducted by the Cancer Dependency Map Project (DepMap) and identified the E3 ligase DCAF5 as a specific vulnerability of RT cells with SMARCB1 loss-of-function. The role of DCAF5 was confirmed by CRISPR-based competitive fitness assays and short hairpin RNA (shRNA)-mediated knockdown, showing that DCAF5 is required for the proliferation of SMARCB1-mutant RT lines, but not for cancer cells harbouring other SWI/SNF mutations. To validate these results, the authors generated an isogenic HEK293T cell model in which endogenous SMARCB1 was knocked out using CRISPR-Cas9 and SMARCB1 could be re-expressed under the control of an inducible promoter. Consistent with data from SMARCB1-mutant RT cells, knockdown of DCAF5 only affected the growth of SMARCB1 deficient HEK293T cells. These experiments suggested that DCAF5 acts as an E3 ubiquitin ligase of SMARCB1-deficient BAF and PBAF, but not of wild-type SWI/SNF. Indeed, DCAF5 knockdown in the isogenic HEK293T cell model increased protein levels of SWI/SNF subunits including ARID1A, SMARCA4, PBRM1 and SMARCC1 but not components of the ncBAF complex such as BRD9. Analysis of the ubiquitinome by di-Gly proteomics confirmed that SMARCA4, ARID1A and SMARCC1 were ubiquitinated specifically in SMARCB1 loss-of-function cells suggesting that these proteins are indeed degraded by the ubiquitin quality control system (Fig. 1).

The strong dependence of SMARCB1-deficient RT cells on DCAF5 suggests that it is not SMARCB1 deficiency that drives tumorigenesis in RT, but the DCAF5-dependent degradation of BAF and PBAF complexes, which are destabilised by the absence of SMARCB1 and therefore cleared by the ubiquitin quality control pathway. This intriguing hypothesis also suggests that SMARCB1-deficient BAF and PBAF are still functional. To test this, chromatin immunoprecipitation followed by sequencing (ChIP-seq) was performed. As proposed by this working model, ChIP-seq data showed that in DCAF5 knockdown cells, SMARCA4, ARID1A and SMARCC1 occupancy was increased at sites where wild-type SWI/SNF would normally bind, thus depletion of DCAF5 rescued deficiency of the tumour suppressor SMARCB1 in RT. Excitingly, DCAF5 depletion not only rescued the SMARCB1-deficient loss of SWI/SNF binding at enhancers, but also restored histone marks such as H3K27ac and H3K4me1 at active enhancers a function that has been associated with SMARCB1. As expected, the restoration of H3K27ac marks was due to increased protein levels of the histone acetyltransferase p300 and co-localisation of this enzyme with SWI/SNF complexes at lineage-specific enhancers known to be modulated by SMARCB1. Furthermore, SMARCB1-deficient BAF and PBAF complexes were still active as chromatin remodellers, as inhibition of SMARCA2/4 ATPases by the small molecule inhibitor BRM014 rescued the effect of DCAF5 knockdown. Interestingly, the study showed that the chromatin remodelling activity of SMARCB1-deficient SWI/SNF was still functional enough to restore chromatin accessibility to transcription factors such as AP1, which depend on SWI/SNF activity.

The study by Radko-Juettner et al. provides exciting new insights into SWI/SNF function in SMARCB1-deficient tumours and provides a new perspective on the tumour suppressor role of SMARCB1. The identification of DCAF5 as an E3 ligase that specifically degrades SMARCB1-deficient SWI/SNF subunits also makes a compelling case for targeting the WDR domain of DCAF5 as a potential treatment strategy for aggressive childhood cancers with SMARCB1 loss of function, the second leading cause of childhood cancer death. This therapeutic strategy is supported by the *Dcaf5* knock-out mouse, which does not show a pathological phenotype. The structural model of the DCAF5-DDB1(ΔB1)-DDA1 complex provided by the authors using cryogenic electron microscopy (cryo-EM) will serve as a structural model for the rational design of such ligands.
